# Recurrent Scrotal Fibroepithelial Polyp in a 7‐Month‐Old Infant: A Case Report and Literature Review

**DOI:** 10.1002/ccr3.70683

**Published:** 2025-08-07

**Authors:** Meng Gui, Qingbao He, Lei Zhang

**Affiliations:** ^1^ Department of Minimally Invasive Urological Surgery Children's Hospital Affiliated to Shandong University Jinan China; ^2^ Department of Minimally Invasive Urological Surgery Jinan Children’s Hospital Jinan China

**Keywords:** electrocautery, fibroepithelial polyp, infant, recurrence, scrotum

## Abstract

Scrotal fibroepithelial polyps (FEPs) in infants are extremely rare and can be misdiagnosed as common skin conditions. Complete surgical excision is essential to prevent recurrence, as incomplete removal leads to regrowth. Adjunctive electrocautery is effective in eliminating residual polyp tissue, reducing recurrence risk, and ensuring optimal long‐term outcomes.

## 1 Introduction

Fibroepithelial polyps (FEPs), also known as soft fibromas or skin tags, are benign mesenchymal tumors affecting cutaneous and mucosal tissues. In the pediatric population, scrotal FEPs are exceptionally rare. Although well‐documented in adults and in urologic sites such as the ureter and bladder, their presentation as recurrent, pedunculated scrotal lesions in infants has not been extensively studied. Kubelis‐López et al. previously described a congenital scrotal FEP in a 3‐month‐old boy [1], but the mechanisms of recurrence and optimal management strategies remain unclear.

Previous studies have documented FEPs in various anatomical locations. Lozano‐Peña et al. [2] and Bahadur et al. [3] reported vulvar FEPs in young women, which grew over years into large polyps. Kampantais et al. [4] described a case of malignant transformation within a fibroepithelial polyp of the glans penis. More recently, Zhanghuang et al. [5] reported a congenital giant scrotal FEP in a 9‐month‐old infant, which had rapidly enlarged and even ruptured before surgical intervention. Complete excision resulted in a favorable outcome with no recurrence at 6 months postoperatively. These reports underscore the importance of early recognition and complete surgical excision in preventing complications and recurrence.

We report a case of a 7‐month‐old infant with recurrent scrotal FEP, emphasizing the diagnostic challenges, surgical management considerations, and the role of electrocautery in preventing recurrence.

## 2 Case History and Examination

**FIGURE 1:**
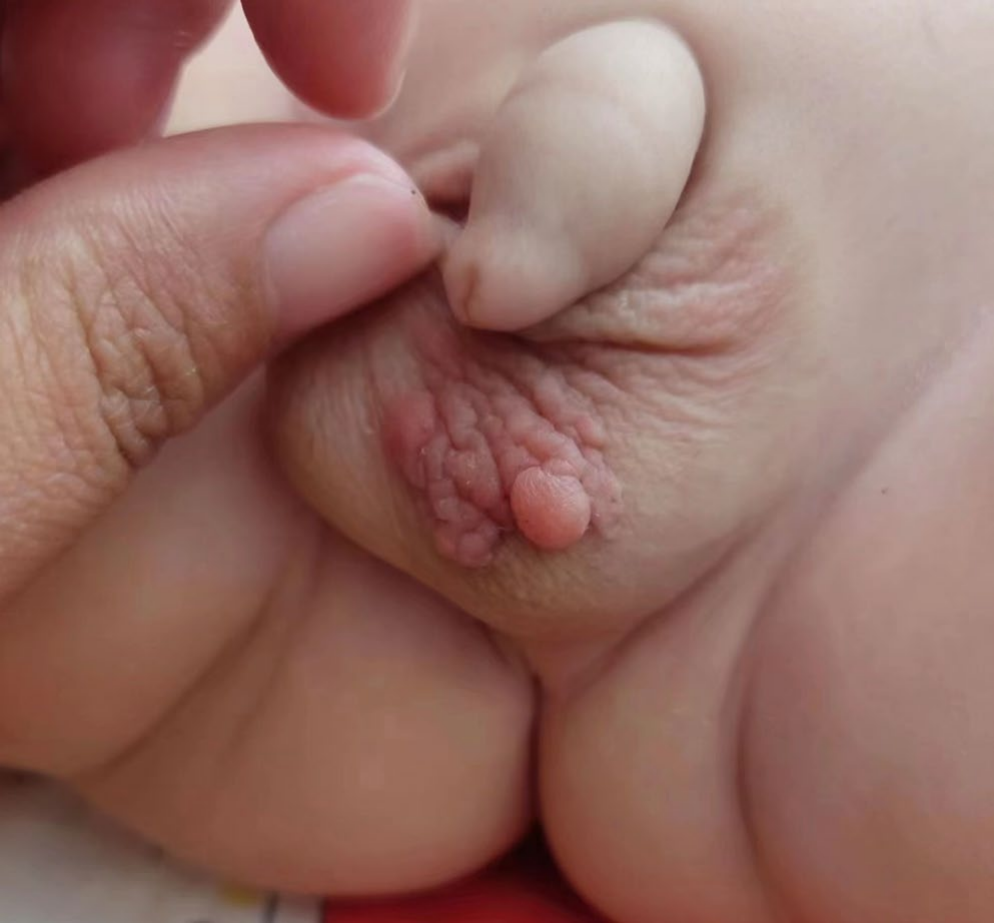
Initial presentation of the scrotal fibroepithelial polyp in the 7‐month‐old infant. Multiple smooth, non‐tender nodules up to 4 cm in diameter are seen extending across the scrotal midline and attached by a pedunculated base.

A 7‐month‐old male infant was brought to our hospital with a left scrotal swelling that was first noticed at 2 months of age. Initially, the lesion was misdiagnosed as eczema and treated conservatively, with no improvement. On examination at 7 months, multiple smooth, non‐tender nodules were observed on the left scrotal skin, extending across the midline and anchored by a pedunculated base. The largest nodule measured approximately 4 cm in diameter (Figure 1). The child exhibited no systemic symptoms or distress.

## 3 Differential Diagnosis, Investigations, and Treatment

### 3.1 Differential Diagnosis

- Hemangioma.
- Verrucae.
- Benign cutaneous tumor (e.g., neurofibroma, lipoma).
- Malignant lesions (rare in infancy).

Given the lesion's pedunculated morphology and growth pattern, an excisional biopsy was planned.

### 3.2 First Surgery

**FIGURE 2:**
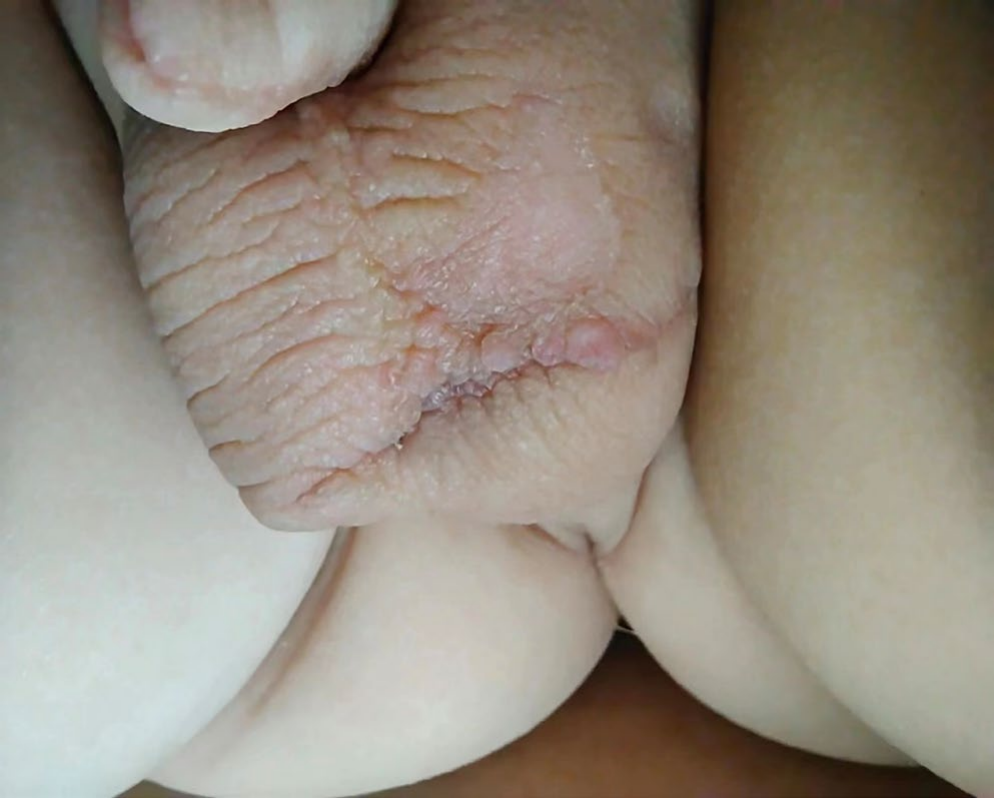
Intraoperative view during the first surgery (June 15, 2021). A spindle‐shaped skin excision was made at the base of the lesion, and a monopolar high‐frequency electrocautery device was used on the lesion surface for hemostasis and ablation of residual tissue.

On June 15, 2021, a spindle‐shaped skin excision was performed, including the base of the pedunculated lesion. Intraoperatively, high‐frequency monopolar electrocautery was applied for hemostasis and to treat smaller residual lesions. Postoperatively, the excised lesion appeared as shown in Figure 2, demonstrating the typical gross morphology. The histopathological examination confirmed FEP, characterized by fibrovascular stroma covered with stratified squamous epithelium.

### 3.3 Recurrence and Second Surgery

**FIGURE 3:**
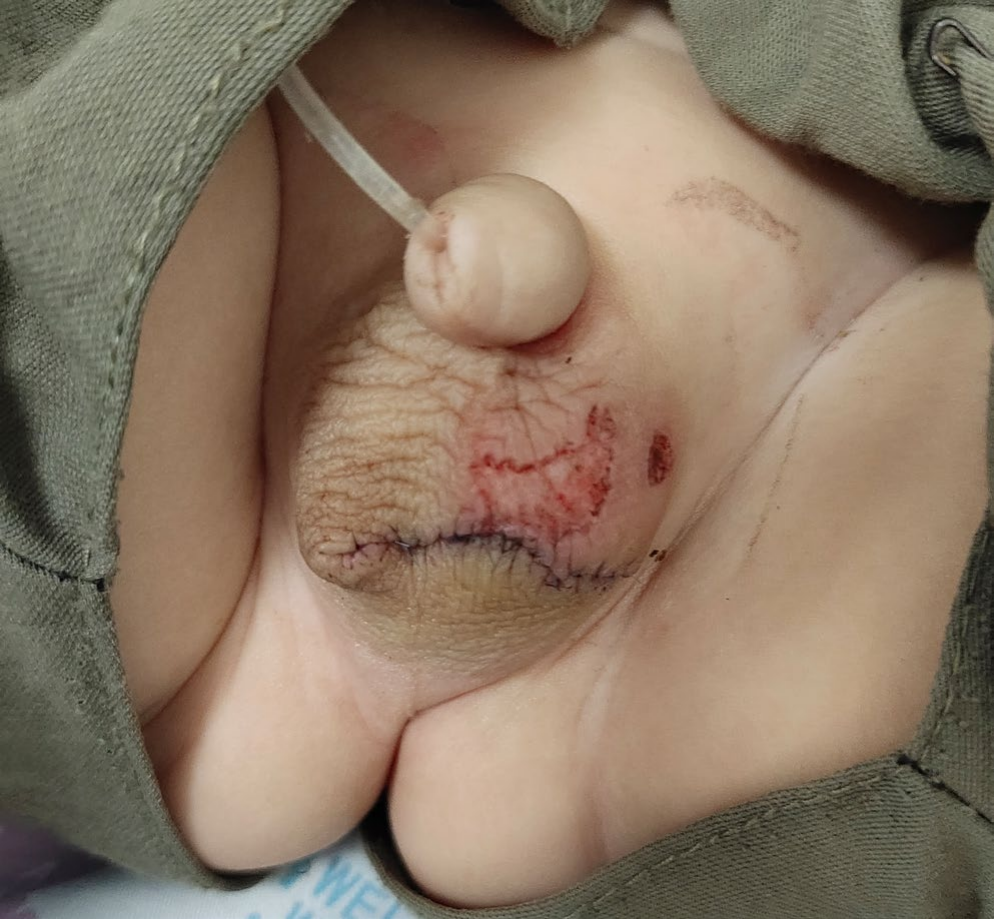
Appearance of the scrotum 1 month after the initial surgery. Several pedunculated red nodules (recurrent polyps) have reappeared along the surgical scar line, and some flatter lesional skin areas persist above the incision.

Within 1 month, multiple pedunculated masses reappeared along the previous surgical margin, indicating recurrence (Figure 3). Several smaller, non‐pedunculated skin lesions persisted above the incision line. In an outpatient setting, low‐energy monopolar electrocautery was applied to these residual superficial lesions, resulting in flattening of the affected skin area.

**FIGURE 4:**
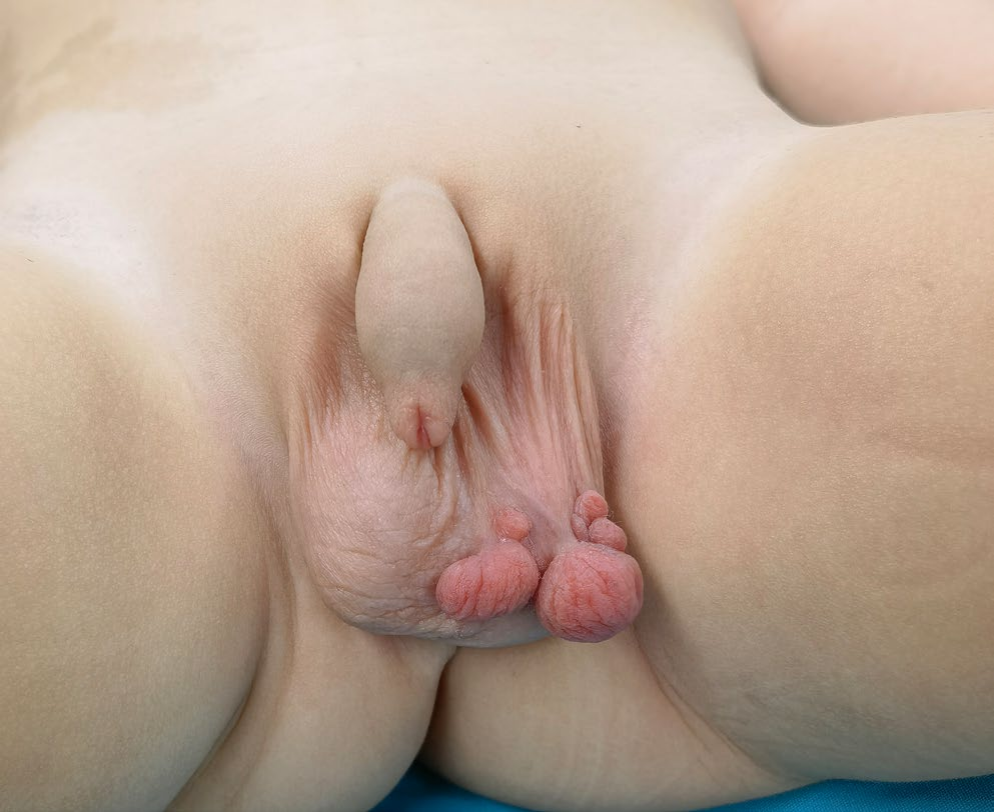
Intraoperative view during the second surgery (May 11, 2022). The recurrent lesion was excised longitudinally along its base, and adjacent tissue was treated with targeted electrocautery. The lesion's morphology was similar to the initial FEP.

**FIGURE 5:**
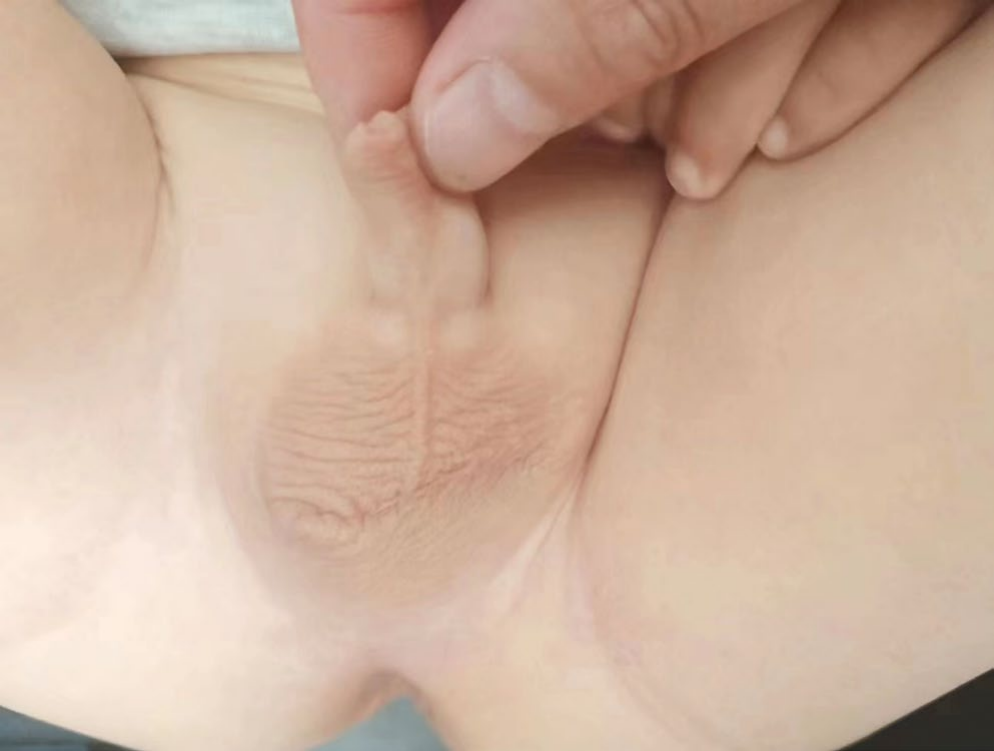
Follow‐up examination 6 months after the second surgery, showing a well‐healed scrotum with no residual lesions or recurrence of the fibroepithelial polyp.

A second surgery was performed on May 11, 2022, due to the continued presence of recurrent pedunculated polyps. This procedure involved a longitudinal excision along the base of the recurring lesions (Figure 4). Any visible abnormal tissue was widely excised, and electrocautery was again utilized to cauterize adjacent tissue margins. Histopathology reaffirmed the initial diagnosis of FEP. Postoperative recovery was uneventful, and at 6 months postoperatively, the surgical site had healed well with no recurrence or new lesions (Figure 5).

## 4 Conclusion and Results (Outcome and Follow‐Up)

At 6 months postoperatively, the surgical site remained well‐healed, with no residual lesions or recurrence. This case underscores the importance of thorough excision and suggests that electrocautery is a useful adjunct to prevent recurrence of scrotal FEPs in infants.

## 5 Discussion

Fibroepithelial polyps are benign tumors of mesodermal origin. They typically manifest as pedunculated, papillomatous growths consisting of an epidermal covering and a fibrovascular core. While commonly observed on the skin, FEPs in mucosal locations (such as the uroepithelium) are relatively uncommon [6].

Scrotal or perineal FEPs are exceedingly rare, particularly in male infants. Previous reports have documented cases of vulvar FEPs in young women [2, 3], with lesions growing over years into large polyps. The etiology of FEPs remains uncertain, but hormonal influences, metabolic factors, and chronic irritation have been proposed as contributing factors [7]. Malignant transformation is extremely rare but has been reported in isolated cases [4].

Surgical excision remains the mainstay of treatment. Cutaneous FEPs, such as those in our patient, are generally cured with simple excision, although wide excision may be necessary for larger lesions. Incomplete initial resection in this case resulted in recurrence, necessitating a second surgery. We found that adjunctive monopolar electrocautery effectively prevented further regrowth, marking the first documented case of its successful use for scrotal FEPs.

For ureteral FEPs, minimally invasive endoscopic resection with holmium or thulium laser ablation has shown good outcomes [8]. Similarly, monopolar electrocautery has been used in endourological management, such as in ureteral polypectomy via a Bugbee electrode [9].

Early and accurate diagnosis is critical in preventing mismanagement. In this case, initial treatment for presumed eczema delayed definitive intervention. Recognizing the characteristic appearance of scrotal FEPs can help clinicians avoid unnecessary delays in treatment.

Overall, the prognosis for FEPs is excellent following complete excision. While recurrence in our patient resulted from incomplete removal rather than aggressive behavior, long‐term follow‐up remains essential to monitor for potential regrowth.

## Author Contributions

**Meng Gui:** project administration, writing – original draft, writing – review and editing. **Lei Zhang:** supervision, writing – original draft, writing – review and editing. **Qingbao He:** writing – review and editing.

## Consent

Written informed consent was obtained from the patient's legal guardian for the publication of this case report and accompanying images.

## Conflicts of Interest

The authors declare no conflicts of interest.

## Data Availability

All relevant data supporting the findings of this case report are included within the article. No external datasets were generated or analyzed during the study.
